# Global statistical regularities modulate the speed of visual search in patients with focal attentional deficits

**DOI:** 10.3389/fpsyg.2014.00514

**Published:** 2014-06-12

**Authors:** Lucilla Lanzoni, David Melcher, Gabriele Miceli, Jennifer E. Corbett

**Affiliations:** ^1^Center for Mind/Brain Sciences, University of TrentoRovereto, Italy; ^2^Center for Neurocognitive Rehabilitation, University of TrentoRovereto, Italy; ^3^Cognitive Neuropsychology Lab, Harvard UniversityCambridge, MA, USA

**Keywords:** mean size representation, perceptual averaging, ensemble statistics, visual neglect, visual search, attention

## Abstract

There is growing evidence that the statistical properties of ensembles of similar objects are processed in a qualitatively different manner than the characteristics of individual items. It has recently been proposed that these types of perceptual statistical representations are part of a strategy to complement focused attention in order to circumvent the visual system’s limited capacity to represent more than a few individual objects in detail. Previous studies have demonstrated that patients with attentional deficits are nonetheless sensitive to these sorts of statistical representations. Here, we examined how such global representations may function to aid patients in overcoming focal attentional limitations by manipulating the statistical regularity of a visual scene while patients performed a search task. Three patients previously diagnosed with visual neglect searched for a target Gabor tilted to the left or right of vertical in displays of horizontal distractor Gabors. Although the local sizes of the distractors changed on every trial, the mean size remained stable for several trials. Patients made faster correct responses to targets in neglected regions of the visual field when global statistics remained constant over several trials, similar to age-matched controls. Given neglect patients’ attentional deficits, these results suggest that stable perceptual representations of global statistics can establish a context to speed search without the need to represent individual elements in detail.

## INTRODUCTION

Visual cognition involves processing both the global aspects of the scene, such as the overall gist and layout, and specifically focusing attention on individual objects to guide actions. Gombrich (1979) introduced the term “etcetera principle,” referring to the perception of global patterns in natural scenes. The extraction of scene meaning and predictions about what is likely to be present in the scene are facilitated by the structure and redundancy inherent in natural scenes (Kersten, 1987), and by the observer’s familiarity with the typical events and objects associated with the given environment (Gombrich, 1979; Melcher and Colby, 2008). In attempt to cope with severe capacity limitations, the visual system could use this inherent redundancy to form compressed perceptual representations of the world, treating objects that share similar properties as a group, or “ensemble” (Ariely, 2001; Alvarez, 2011; Corbett and Melcher, 2014).

Ariely (2001) was the first to demonstrate that the mean size of an ensemble of heterogeneously sized elements is encoded with much greater precision than the sizes of the individual elements comprising the set. Based on these results, Ariely (2001, 2008) proposed that the visual system forms a simplified representation of sets of multiple objects sharing salient features in order to free limited capacity attentional resources. This set representation does not include precise information about single item identity, but instead represents the overall average properties of the set. In support of this proposal, Chong and Treisman (2003) found that observers were as accurate at determining which of two displays of heterogeneously sized circles had the larger average size as they were at determining which of two displays of homogeneously sized circles had the larger size, and which of two single circles was larger. Several other stimulus properties appear to be statistically represented more precisely than information about individual elements, such as average orientation (Dakin and Watt, 1997; Parkes et al., 2001), average speed (Watamaniuk and Duchon, 1992), average direction of motion (Watamaniuk et al., 1989), and even higher-level abstract properties, such as emotional expression (Haberman and Whitney, 2007). Taken together, these results provide strong evidence that the average properties of a set of similar objects can be extracted with as much, or even more precision than information about the characteristics of the individual elements in the set. Encoding the average of a set of potentially noisy measurements allows for a more precise representation of the set’s average, free of uncorrelated error between individual measurements. More broadly, representing the statistical properties of sets, such as the average (or any other “prototypical value”) allows for a more coherent representation that, despite simplification, is sufficient to navigate in the real world (Ariely, 2001).

## PERCEPTUAL AVERAGING AND ATTENTION

There is mounting evidence that representing individual items involves qualitatively different processes than computing means. Although the detailed representation of a few salient objects requires focal attentional resources, it has been proposed that the mechanisms responsible for statistical representations of ensemble properties can operate under less demanding conditions of “diffuse” or “global” attention. For example, when observers distributed attention broadly over a set of items, they were at chance to recall the sizes of individual members of the set, but still retained a precise representation of the set’s mean size (Chong and Treisman, 2005). Several other studies have demonstrated that even items that are masked (Choo and Franconeri, 2010), or crowded (Parkes et al., 2001) from conscious awareness, or items that are presented when focal attentional resources are not available for detailed encoding (Joo et al., 2009; Corbett and Oriet, 2011) are nonetheless included in the calculation of average size. Overall, empirical evidence is consistent with the existence of two qualitatively different processes: one devoted to processing individual objects in detail using focused attention (with a capacity of around 3–4 elements at a time), and the other involved in representing the more global properties of sets of similar objects and the overall gist or meaning of the scene. This second mechanism would provide an effective way of dealing with capacity limitations of visual attention and visual working memory, as individual elements’ identities are not included in or necessary to form this global set representation.

## NEUROLOGICAL PATIENTS AND PERCEPTUAL AVERAGING

Perhaps the most convincing evidence that perceptual averaging can occur without focused attention is given by studies from neurological patients with noted attentional deficits. Demeyere et al. (2008) first examined statistical processing in a simultanagnosic patient, G.K. Simultanagnosia is described as a difficulty in perceiving more than one or two objects at a time. Despite G.K.’s inability to consciously attend more than one object at a time, when asked to report whether a test probe was a member of a previously shown set, he incorrectly identified the mean size or shade (never actually shown) of a set of two classes of exemplars more often than when only one class was present in the set. These results suggest that even though G.K. could not attend to more than one object at a time, he still automatically averaged the two classes of items present in the display and incorrectly chose the probe that corresponded to the average never actually present in the sets.

In addition to these results demonstrating statistical averaging in a simultanagnosic patient, studies examining summary representations in neglect patients provide further evidence for statistical extraction without focused attention. Visual neglect (or hemispatial neglect) is a condition that typically occurs following an acute cerebrovascular accident in the right hemisphere (for reviews, see Vallar and Perani, 1987; Mort et al., 2003), and is characterized by a failure to attend to objects located in the contralesional side of space in the absence of primary sensory deficits. Although neglect has been interpreted by some as a failure to integrate perceptual and sensory information (e.g., De Renzi, 1982), or as failure to correctly form internal representations of space (e.g., Bisiach and Berti, 1987), the most widely accepted interpretation ascribes neglect to a deficit in spatially orienting attention towards stimuli in the contralesional field (e.g., Kinsbourne, 1993). However, not all symptoms of visual neglect are lateralized to the contralesional field. Critically, visual neglect also involves spatially non-lateralized mechanisms resulting in a fundamental loss of attentional capacity throughout the visual field, not specific to a particular hemifield or region of space (for reviews, see Robertson, 2001; Husain and Rorden, 2003).

A growing body of evidence indicates that some kind of global processing still occurs in the neglected regions of the visual field, despite the lack of local awareness (for review see: Driver and Vuilleumier, 2001). The findings described above that observers automatically extract statistical representations of collections or ensembles of objects with little or no demands on focal attentional resources suggest that patients with compromised attentional resources may still retain the ability to extract global statistical properties in neglected regions. Recent studies have shown evidence that patients are indeed sensitive to summary statistics within the neglected regions, despite their inability to perceive the individual elements comprising the summarized sets. Pavlovskaya et al. (2010) first reported that unilateral neglect patients extract statistical properties of a visual scene. They suggested a weighted average computation across hemifields, which relies more on the information located on the right (spared) side compared to the information located on the left (neglected) side.

Most recently, Leib et al. (2012) investigated whether neglect patients were equally sensitive to the average properties of elements in both neglected and spared regions. Patients were presented with a single circle target and subsequently instructed to search for the same target in display of multiple circles on one side of the screen, ignoring a set of distractor triangles located on the other side of the screen. In target absent trials, three of the four patients made more false alarms, incorrectly reporting that the target was present when it was the mean of the circles in the neglected region. Conversely, when the circles were on the right, all patients made more false alarms in reporting that a target that was the mean of the entire set of triangles and circles was present, providing additional evidence that, although not consciously perceived, the information on the left side (distractors) was being pooled in the computation of average size. Interestingly, these findings that information in the neglected hemifield was not only averaged, but also interfered with the averaging of information in the spared hemifield suggest that neglect patients might rely even more on global processing than control participants.

Given these studies demonstrating that patients are sensitive to the average characteristics of stimuli within the neglected region, it is possible that these statistical properties are used to establish a stable global context to facilitate patients’ interactions within the neglected areas. Along these lines, Saevarsson et al. (2008) demonstrated that a stable spatial context improved patients’ performance in a visual search task. When patients searched for a target stimulus with a repeated vs. new spatial relationship to the background context, they demonstrated the greatest benefits of contextual cueing (in terms of faster reaction times) for targets presented in the neglected hemifield, in agreement with Chun and Jiang’s (1998) findings for healthy participants. These results demonstrate that contextual cueing by perceptual grouping can occur even in the neglected hemifield, and suggest that this type of perceptual organization may occur before attentional processing, at lower levels in the perceptual hierarchy.

## CONTEXT AND MODELS OF VISUAL SEARCH

Similar to Chun and Jiang (1998) and Saevarsson et al.’s (2008) findings that the repeated spatial arrangement between a set of colored contextual elements and a target can speed visual search, several other studies have demonstrated such benefits of repeated spatial context using natural scenes (e.g., Hidalgo-Sotelo et al., 2005; Brockmole and Henderson, 2006a,b; Oliva and Torralba, 2006). These findings can be explained in terms of Torralba et al.’s (2006) Contextual Guidance model of attention in which image locations with salient features are defined in a feed-forward, pre-attentive manner. In this framework, it is not necessary to first segment the scene into objects, but instead, the global statistics of the image are used to guide attention and predict where salient objects and events will likely occur. Findings that visual search is facilitated by global structural and statistical redundancies can also be interpreted in the context of the Guided Search 2.0 model of attention (Wolfe, 1994). In this view, context is built over successive presentations of the same scene properties and integrated into a persistent bottom-up feature map. As this map stabilizes over several successive presentations of the same properties, the target is more easily localized. Importantly, the two models outlined are not representative of an exhaustive list of the models that have been developed to account for performance in visual search tasks. Contextual cueing results are consistent with numerous accounts of visual search. We have included an outline of these two particular models because they can straightforwardly illustrate how the repeated context of a scene’s background can act to compress the inherent redundancy in incoming visual information and direct processing resources to predicted locations of salient targets and events.

## GOAL OF THE PRESENT STUDY

Findings from studies of both neurotypical observers (e.g., Chun and Jiang, 1998) and neurological patients (Saevarsson et al., 2008) that a repeated spatial relationship between a target and a given context can facilitate visual search and that global set properties are perceptually encoded (e.g., Leib et al., 2012) raise the possibility that the mere repetition of global statistical regularities may similarly facilitate search, regardless of the spatial relationship between the target and the contextual elements. Neglect patients serve as an interesting test group for studying the possible effects of global visual processing in the near absence of focused attention (or at the very least, under conditions in which focused attentional resources are severely compromised in the neglected regions). Therefore, we conducted the present investigation to examine how repeating the global ensemble statistics of the background may also affect the speed of patients’ visual search in neglected as compared to spared regions, even when there is no predictable spatial arrangement of the context linked to the location of the target.

We modulated the statistical stability of the context by changing the mean size of background elements after several trials, and measured the speed with which patients correctly discriminated the tilt of a target element in neglected and spared regions of the visual field. Importantly, the local sizes of individual elements changed on every trial, and only the global mean size of the entire display remained constant over several trials. If the stability of the mean size of the background elements can be used to construct a global representation of the scene despite continuous changes in local elements, this should help to eliminate the need to represent each element in detail and free processing resources for visual search in the neglected regions. We also tested the effects of global statistical stability in left and right hemifield locations of age-matched controls to help ensure that any observed differences in patients’ neglected and spared regions could not otherwise be accounted for by left-right processing asymmetries that might have emerged in older adults. A preserved ability to represent the global statistical properties of visual scenes may help to explain findings that patients with severe attentional deficits nonetheless experience a stable and coherent visual world.

## MATERIALS AND METHODS

### PARTICIPANTS

Three patients with chronic unilateral left neglect (all males) were recruited from The University of Trento’s Center for Neurocognitive Rehabilitation (CeRiN). All three patients were more than 1-year post-cerebral injury at the time of testing, had mild visual neglect, and participated in one testing session which lasted approximately 60–90 min in total. In addition, we tested a group of 12 age-matched control participants (all male, mean age = 60.92 years), each in a single session that lasted approximately 30–45 min in total. For both patients and controls, vision was normal or corrected-to-normal and all were right-handed. The University of Trento’s Institutional Review Board approved all procedures.

### PATIENTS

All patients were referred to us by the University of Trento’s Center for Neurocognitive Rehabilitation (CeRiN) after having been diagnosed as having mild chronic neglect by at least one neurologist, and each completed several standard cancelation and line bisection tasks for spatial neglect at least 1 year prior to participating in the present study in July of 2013.

### BELL CANCELATION TASK (adapted from Gauthier et al., 1989)

As part of visual neglect diagnostic and assessment protocol at CeRiN, all patients completed several bell cancelation tests. During each test, patients were presented with a white sheet of paper containing 315 black silhouettes of various objects (e.g., house, horse, bell…), with a total of 35 bell targets distributed randomly such that there were 17 bells on the right of the sheet and 18 on the left. After insuring patients could name all of the stimuli displayed in isolation, they were presented with the sheet containing the 315 items and instructed to draw a line through all of the bells as quickly as possible without moving the torso or realigning central posture relative to the sheet. The top portion of **Table 1** displays the total number of bells detected on the right and left sides for each patient, as well as the total time each patient required to complete the task (e.g., Vallar et al., 1994) during the first testing session after the initial diagnosis of visual neglect at CeRiN, as well as during the most recent test prior to the present investigation.

**Table 1:** Patients’ performance on the bell cancelation (e.g., Gauthier etal., 1989; Vallar etal., 1994) and line bisection tasks (e.g., Rode etal., 2006) after the date of their initial diagnosis at CeRiN, as well as their most recent scores on each task.

	Patient 1	Patient 2	Patient 3
**Bell cancelation** (Gauthier et al., 1989; Vallar et al., 1994)
Date of test at initial diagnosis	February, 2012	September, 2011	October, 2011
Right (out of 17)	16	15	16
Left (out of 18)	18	6	17
Total time (min)	150	128	170

Date of most recent test	August, 2013	October, 2012	May, 2013
Right (out of 17)	16	14	16
Left (out of 18)	17	14	18
Total time (min)	121	91	178
**Line bisection** (Rode et al., 2006)
Date of test at initial diagnosis	February, 2012	September, 2011	October, 2011
Average % deviation to right of center in 10, 15, and 25 cm lines	3.4	13.8	4.2

Date of most recent test	August, 2013	October, 2012	October, 2013
Average % deviation to right of center in 10, 15, and 25 cm lines	-0.2	0.99	1.6

### LINE BISECTION TASK (adapted from Rode et al., 2006)

Patients also performed several line bisection tasks over the course of their diagnosis and treatment at CeRiN. During each test, patients were presented with a sheet of paper containing a single horizontal line subtending 10, 15, or 25 cm and asked to draw a line through the center of the line. Each line length was presented separately to each patient in random order. The average percentage of deviation to the right of center relative to the total length of the line was calculated for each patient. The scores and dates of testing after the initial diagnosis at CeRiN, as well as these values for the most recent test prior to the present investigation are presented at the bottom of **Table 1**.

#### Patient 1 (age 51)

**FIGURE 1:**
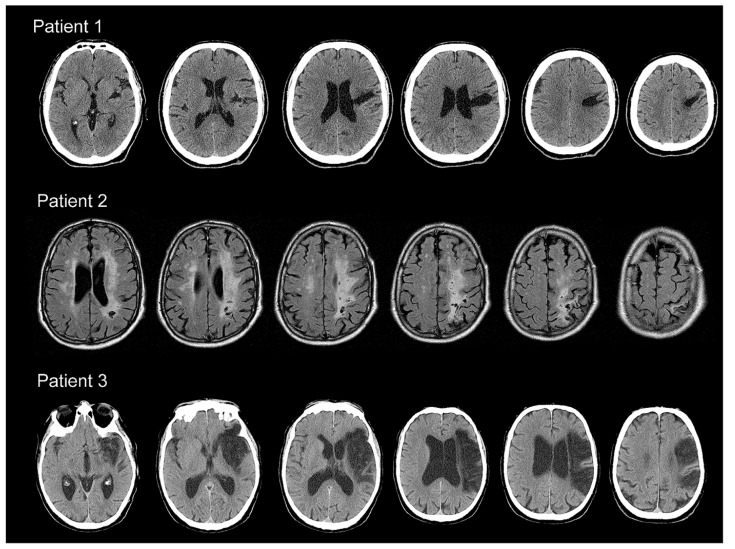
**Top:** consecutive slices of CT scans for Patient 1 illustrating right frontal, temporal, and parietal lesions. **Middle:** consecutive MR slices of scans for Patient 2 showing damage to the right frontal, parietal, and occipital regions. **Bottom:** consecutive slices of CT scans for Patient 3 showing widespread lesions over the right frontal, parietal, and temporal cortices.

In 2011, Patient 1 suffered from right frontal, temporal, and parietal hemorrhages, with damage extending to subcortical white matter. A diagnosis of unilateral left neglect was issued by a neurologist at CeRiN 1 month after the lesion, and confirmed 6 months later by a different neurologist. In 2012, Patient 1 was involved in a 6-month neurocognitive rehabilitation program at CeRiN, aimed at enhancing attention and improving visual search. Speech therapy and motor therapy were concurrently undertaken at the time of the present experiment. Because Patient 1 was noted to be claustrophobic, only CT scans were available for inclusion in **Figure 1** (Top). Following treatment after the acute phase of neglect, Patient 1 showed marked improvement and scored within the normal limits of formal testing in the bell cancelation and line bisection tasks presented in **Table 1**. However, both a clinical psychologist and a neurologist at CeRiN retained his diagnosis of mild chronic left neglect based on qualitative assessments. For example, when asked to imagine the Italian peninsula from Sardinia and list the regions he could see from this imaginary spot, on three separate occasions he either omitted Liguria (the leftmost region), or switched the relative position of Liguria and Tuscany (the two leftmost regions). On double simultaneous visual stimulation, he showed left-sided omissions when both hemifields were stimulated, and omitted stimuli in the relative left when both hands of the examiner were in his left visual field.

#### Patient 2 (age 69)

Patient 2 was diagnosed with hemispatial neglect in 2011 by a neurologist at CeRiN following a vascular encephalopathy that involved right fronto-parietal cortical and subcortical regions, illustrated in the MR scans in the middle of **Figure 1**. Following the ischemic event until the time of our study, Patient 2 had been engaged in several rehabilitation programs, aimed at improving visual exploration and lateralized visuo-spatial attentional deficits. Prismatic adaptation was implemented for the specific treatment of visuo-spatial neglect (see Redding and Wallace, 2006 for a review). Constrained Induced Movement Therapy (see Wolf et al., 2002 for a review) was later performed to increase the use of the affected upper limb. At the time of the present investigation, Patient 2 was not involved in any rehabilitation programs. As shown in **Table 1**, Patient 2 improved in the bell cancelation and line bisection tasks over the course of treatment, but both recent tests still showed evidence of left hemispatial neglect despite therapeutic efforts.

#### Patient 3 (age 74)

Patient 3 was diagnosed with left occipital and thalamic ischemia in 2008, which relapsed in a new vascular event in 2009. Visuo-spatial neglect was diagnosed by a neurologist at CeRiN following the second infarct, which involved the right middle cerebral artery, resulting in damage in frontal, temporal, and parietal cortices, as well as subcortical structures. As Patient 3 had a cardiac pacemaker, only CT scans illustrating the relevant cerebral damage were available for inclusion in **Figure 1** (Bottom). Since 2009, Patient 3 had been involved in several rehabilitation programs aimed at improving visuo-spatial attention, visual memory, and visual cognition in general. Prismatic adaptation was also performed as a specific treatment of visuo-spatial neglect. At the time of the present experiment, Patient 3 had recently begun a new cycle of cognitive therapy. As illustrated in **Table 1**, Patient 3 improved on the bell cancelation task over the course of treatment, but still showed evidence of left hemifield neglect in the line bisection task during the most recent testing session. Importantly, a neurologist and a clinical psychologist at CeRiN noted that Patient 3 sometimes showed signs of neglect throughout the visual field, not always restricted to the left hemifield, likely resulting from the widespread lesions incurred over the two ischemic events between 2008 and 2009.

### ASSESSMENT OF NEGLECT IN PATIENTS FOR EXPERIMENT-SPECIFIC LOCATIONS

Traditional paper-and-pencil or touch-screen assessments of neglect (e.g., Bell Test; Gauthier et al., 1989) were not able to measure performance in the specific stimulus locations used in our main experiment, nor were they free of left-right/top-bottom search strategy confounds observed in pilot studies, likely strengthened by the visual therapies typically undergone by neglect patients. It has been repeatedly demonstrated that patients with minimal evidence of neglect on traditional measures (like typical cancelation and line bisection tasks used to assess patients in the present investigation) nonetheless exhibit notable impairments in more precisely controlled adaptive computerized tasks, such as conjunctive search (e.g., List et al., 2008) and dual-task paradigms (e.g., Bonato et al., 2013) simulating attentional demands more similar to those of real-world interactions. Furthermore, as previously noted, visual neglect also involves a fundamental loss of attentional capacity throughout the visual field (for reviews, see Robertson, 2001; Husain and Rorden, 2003). Such non-lateralized spatial deficits were apparent in Patient 3, who was reported to occasionally demonstrate widespread attentional deficits in addition to left hemifield neglect. Therefore, in addition to the standard line bisection and cancelation tasks used by neurological professionals to diagnose the three patients with visual neglect (**Table 1**), we developed a detection task to assess the extent of neglect at each of the 64 specific stimulus locations in the main experiment, individually for each patient. Patients performed this additional detection task during the same session as, and immediately prior to the main experiment. Importantly, beyond traditional measures, our detection task allowed for empirical assurance that specific stimulus locations defined as “neglected” in our main experimental task were indeed locations where individual patients exhibited attentional deficits, and regions defined as “spared” showed no such empirical deficits in performance.

Stimuli in this detection task were single Gabors subtending 1° of visual angle with a spatial frequency of four cycles per degree presented in isolation at 50% contrast on a gray background and tilted +45 or -45° from vertical, determined at random on each trial (the same as the target in the main experiment). The detection assessment was divided into three approximately 2-min blocks of 64 trials to allow patients to take regular breaks, and to help ensure they maintained the correct posture within the chin-and-head rest. In each block, each trial began with a 1° white fixation cross on an otherwise blank gray screen. Patients were instructed to fixate on the cross and press spacebar when they were ready to start the trial. Next, the fixation cross turned black and a target Gabor appeared at one of the 64 locations, determined pseudo-randomly on each trial such that each of the 64 locations was tested once over the course of each of the three assessment blocks, for a total of three trials per location over the course of all three blocks. After a brief duration, determined individually for each patient, the target Gabor disappeared and the fixation cross turned red, signaling the patient to respond to the left/right tilt of the target Gabor as in the main experimental task. Patients were instructed to guess if they had not seen the target Gabor. Before beginning the formal assessment of neglect, the experimenter adjusted the duration of the target Gabors for each individual to a constant duration that allowed the specific patient to achieve approximately 75–80% correct performance. This individual-threshold duration was then used for all stimulus locations in the three blocks of the assessment task for the given patient (Patient 1 = 10 ms; Patient 2 = 150 ms; Patient 3 = 100 ms).

Several displays of instructions were shown immediately before the beginning of the assessment task to demonstrate each step, while the experimenter verbally explained the task. Written instructions were lateralized to the right side of the screen as in the main experiment to ensure that patients were able to clearly perceive them during the instructional process. Patients were told that the target had an equal probability to appear anywhere in the screen, and an equal chance of being tilted to the left or the right. Given that breaks were mandatory at the end of each block, and elective breaks could be taken following each trial, patients were required to remain as still as possible within a single trial. They did not begin the main assessment task until the instructions had been fully explained and the experimenter had answered all questions they may have had about the experimental protocol.

**FIGURE 2:**
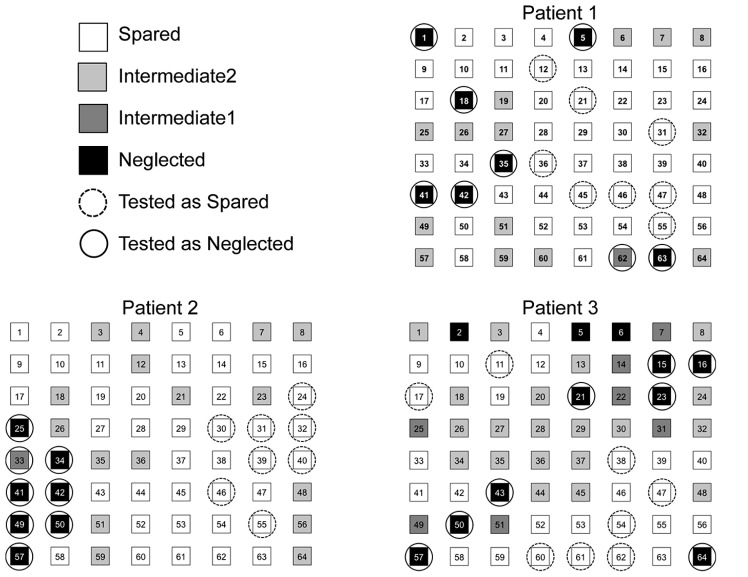
**Maps of Spared locations (white, correctly discriminated 3/3 times), Intermediate2 locations (light gray, correctly discriminated 2/3 times), Intermediate1 locations (dark gray, correctly discriminated 1/3 times), and neglected locations (black, correctly discriminated 0/3 times) resulting from the experimental stimuli-specific location detection task performed by each patient.** For each patient, locations circled with solid lines were used as Neglected locations and locations with dashed circles were used as Spared locations in the main experiment.

The locations mapped by the assessment task were used to define individual patients’ neglected and spared regions in the experimental blocks. Locations for which a given patient correctly discriminated the target’s tilt 3/3 times were labeled as Spared Locations, 2/3 times as Intermediate2 Locations, 1/3 times as Intermediate1 Locations, and 0/3 times as Neglected Locations, with the requirement that each Neglected and Spared set contain at least eight locations. If this requirement was not met, the number of additional locations necessary to yield a total of eight locations in each set was drawn from the corresponding intermediate set of locations (i.e., additional locations were drawn from the Intermediate2 Locations if there were not eight locations in the Spared set and from the Intermediate1 Locations if there were not eight locations in the Neglected set). If there were more than eight locations in either Neglected or Spared set, eight locations were randomly drawn from the set to ensure that every patient searched for the target in an equal number of eight of each of their respective spared and neglected locations. Similarly, eight locations on the left side of the display and eight locations on the right side of the display were randomly selected for each of the twelve left-right hemifield control participants to equate the number of locations with those tested in patients’ neglected and spared regions. The individual maps of these four types of locations and the Neglected and Spared locations chosen for the main experiment for each patient are provided in **Figure 2**.

To further ensure that our detection assessment task measured spatially specific attentional deficits, we also tested three additional age-matched control participants (all male, mean age = 60.33 years, all with normal or corrected-to-normal vision). Control participants with no neural damage or noted attentional deficits should not exhibit the same pattern of consistent impairment in specific locations like those illustrated above for each patient. Such findings would therefore provide further support that the stimulus locations defined as “neglected” for patients in the main experiment task were indeed locations where they exhibited attentional deficits not found for controls. Using the most stringent presentation time of any patient tested (10 ms), we presented the single Gabor in isolation and control participants discriminated the left or right orientation exactly as in the procedure outlined above for patients.

Unlike patients, no control participant missed any location more than once. Control participant 1 missed only one location on one trial, control participant 2 missed 5 locations one time each, and control participant 3 missed 6 locations one time each, whereas all three patients missed at least 7 locations all three times when the stimulus was presented in those respective locations. These three control participants then performed the main experimental task with the target presented in 16 possible locations to equate location uncertainty with the 16 possible locations tested in patients. However, it was not possible to analyze performance in the few locations that controls missed only once in the detection task compared to the disproportionate majority of locations where they never missed the target. Instead, we compared the three control participants’ mean reaction times in the main experimental task in the locations they missed once in the detection task to ±1 standard deviation of their respective mean reaction times in all 16 locations in the main search task (only participant 3 incorrectly indicated the tilt of the target Gabor in the main search task on a single trial. Accuracy was 100% for the other two controls). All mean reaction times for controls’ “missed” locations fell well within one standard deviation of their respective mean reaction times.

Taken together, the findings that control subjects did not perform as neglect patients on the detection task and the finding that control participants’ reaction times in the very few locations that they did miss only once in the detection task did not differ from the mean of their respective reaction times in all 16 possible stimulus locations provide strong evidence that patients in our study did in fact have empirically defined localized attentional deficits that were not exhibited by control participants. Although mapping patients’ detection performance in the specific regions tested in our main search task did not result in a pattern of “classic” left-lateralized neglect, this manner of assigning stimulus locations allowed for added empirical assurance that any effects of manipulating statistical stability for targets in regions defined as “neglected” for patients in our main experimental task were in fact locations where patients exhibited attentional deficits not apparent in their “spared” regions, or observed in control participants.

### MAIN EXPERIMENTAL TASK

All patients and controls participated in the main experiment. On each trial, participants searched an array of horizontally oriented heterogeneously sized Gabors for a target Gabor tilted from vertical, and indicated whether the target was tilted left or right. If the target was tilted to the left, they pressed the left arrow key on a computer keyboard, and if it was tilted right, they pressed the right arrow key.

### APPARATUS

Stimuli were presented on a 23″-inch ACER T230H bmidh monitor, with the screen set to a resolution of 1440 × 900 pixels and a refresh rate of 85 Hz. Responses were recorded on a computer keyboard. Matlab^®^ software (version 2009a), in conjunction with the Psychophysics Toolbox (Brainard, 1997; Pelli, 1997), controlled all the display, timing, and response functions. Participants were tested individually in a dimly lit room, and their heads were stabilized by a chin-and-head rest to ensure that they remained as still as possible at a fixed distance of 57 cm from the monitor on which stimuli were presented for the duration of each assessment and experimental block. At this distance, one degree of visual angle corresponded to 29 screen pixels.

### STIMULI

Stimuli were 64 Gabor patches, with a spatial frequency of four cycles per degree presented at 50% contrast relative to the gray background. The Gabors were arranged in 64 locations within an imaginary 8 × 8 grid. Each individual stimulus location was jittered randomly in the *x*- and *y*-directions by 20 pixels on every trial. On each trial, 64 Gabors were presented, one in each location. All the Gabors had a horizontal orientation varied at random on each trial between 84 and 92° of tilt from vertical in 1° steps, except the target Gabor, which was tilted at random on each trial to the right or left of vertical by 45°. Critically, on each trial, the sizes of the individual Gabors were drawn at random from a normal distribution with a constant standard deviation of 0.15° of visual angle, and one of three means, 0.5, 1, or 1.5° of visual angle, with the exception that the target Gabor always subtended 1° of visual angle. Only the mean size of the entire array of 64 Gabors remained constant for 5–8 trials. Importantly, averaging over all three mean sizes allowed us to examine the effect of manipulating the overall mean size of the Gabors independent of differences in low-level aspects of the displays with the three different mean sizes, such as visibility, luminance, and density. The mean size of the Gabors in each sequence of 5–8 trials was determined pseudo-randomly, such that none of three means were repeated in immediate succession.

### PROCEDURE

Each trial began with the presentation of the 64 Gabors. Participants were instructed to search the display for the only Gabor that was tilted from vertical, and then to indicate whether this target was tilted to the left or right using the corresponding arrows on the computer keyboard. Stimuli remained visible on the screen until a response was given. The stability of the background global statistics was manipulated by changing the distractor Gabors’ mean size. The number of 5–8 stable trials was chosen at random at the start of each sequence in order to prevent subjects from predicting when the mean size of the Gabors would change from a repeating temporal rhythm. Each participant performed two blocks of 24 sequences (approximately 6.5 trials with the same mean size repeated × 24 sequences ~ = 156 trials per block; Patient 1 and all control subjects), or 4 blocks of 12 sequences (approximately 6.5 trials with the same mean size repeated × 12 sequences ~ = 78 trials per block; Patient 2 and Patient 3)^1^.

Prior to the start of the experiment, participants were given written instructions on the computer screen. Instructions were lateralized to the right side of the screen to ensure that patients were able to clearly perceive them during the instructional process. All participants were informed of the length of each block, as well as length of the entire experiment. They were also strongly encouraged not to move their heads or bodies for the entire duration of each experimental block (~6 min), and mandatory breaks were given at the end of each block. Participants were instructed to perform the task as quickly as possible, but never to compromise accuracy, and were informed that their manual reaction times would be recorded. They did not begin the experiment until the experimenter had answered all their questions and ensured they fully understood the experimental protocol. To ensure participants were familiar with the task and could correctly perform it, they each completed one block of 24 practice trials immediately before beginning the experimental blocks. During the practice block, they received verbal feedback about the accuracy of their responses on each trial from the experimenter. Data from this practice block were not analyzed.

## ANALYSIS

Trials were defined as “Stability built” trials if they were the last in a sequence where the mean size had remained constant over several trials (trials 5, 6, 7, or 8 of sequences 5, 6, 7, or 8 trials in length, respectively), and as “Stability broken” trials if they were the first in a new sequence immediately after the mean size had changed. Only such trials in which participants had correctly reported the tilt of the target Gabor were considered for further analysis (>96% of all trials for patients and >95% of all trials for controls), yielding approximately equal numbers of trials in each condition (~48 trials) for each participant. Of these trials, the target was presented pseudo-randomly in the Neglected regions 50% of the time and in the Spared regions the other 50% of the time for patients, and 50% of the time in the left hemifield and 50% of the time in the right hemifield for the twelve age-matched controls. This resulted in approximately 24 trials for each participant in each combination of the Stability (Stability built/Stability broken) and Side (target presented in Neglected or Left/Spared or Right region) experimental conditions.

## RESULTS

**FIGURE 3:**
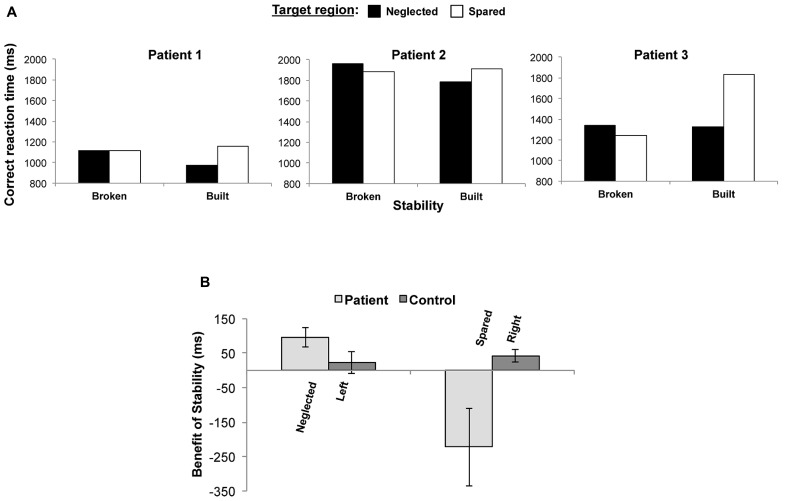
**(A)** Search for target Gabors in neglected regions was facilitated in all three patients when the mean size of the background Gabors remained stable for several trials compared to when the mean size of the background changed and the stable statistical context was broken. **(B)**. The benefit of stability of the background mean size for the twelve age-matched controls was not different across left and right hemifields, nor was the benefit of stability different in patients’ neglected regions and controls’ left hemifields, but there was a significant detriment when the background remained stable and the target was presented in patients’ spared regions compared to when it was presented in the right hemifield for control participants (Error bars represent ± 1 standard error of the mean).

As illustrated in **Figure 3A** and the mean and standard deviation reaction time values displayed in **Table 2**, all patients were faster to make correct tilt judgments in the neglected compared to spared regions when the global statistics of the Gabors remained stable over several trials. Although a 2 (Stability broken vs. built) × 2 (Target in neglect vs. spared region) repeated-measures within-subjects ANOVA for this small sample size did not reveal any significant main or interaction effects [all *F*(1,2)s < 4.820, all *p*s > 0.160] in patients, there was a notably large effect size for Stability ($\eta_{p}^{2}$ = 0.639), as well as the interaction between these factors ($\eta_{p}^{2}$ = 0.707), supporting the consistent effect of stability on search performance in the neglected regions across all patients.

**Table 2:** Mean correct response times and standard deviations (in parentheses) for each patient and the average of the 12 control participants in each of the four experimental conditions, as well as the average correct response times and standard deviations for each patient and for the control group across conditions (right columns), and the group averages for each condition across patients (second row from the bottom) and controls (bottom row).

Subject	Neglect/left stability broken	Neglect/left stability built	Spared/right stability broken	Spared/right stability built	Average over conditions
S1	1111.91 (419.5)	1014.68 (232.2)	1111.82 (322.0)	1154.82 (338.8)	1098.31 (328.19)
S2	1960.56 (1211.2)	1782.48 (1058.4)	1882.14 (845.7)	1914.72 (991.4)	1884.98 (1026.72)
S3	1341.96 (374.1)	1326.42 (427.7)	1241.90 (630.0)	1834.62 (1603.7)	1436.23 (758.93)
Patient average	1471.48 (668.3)	1374.53 (572.8)	1411.95 (599.26	1634.18 (978.0)	1473.17 (704.61)
Control average	961.39 (161.0)	938.12 (116.1)	988.24 (212.0)	945.25 (180.2)	958.25 (167.39)

To examine whether any effects of stable background statistics observed in patients could be due to differences in search performance between the left vs. right hemifields not associated with neglect or aging, we compared the benefit of a stable context on control participants’ search performance for targets in the right vs. left hemifields. For each participant, both patients and controls, we calculated the “benefit of stability” as the difference between the individual’s average correct response times when stability was broken minus when stability was built in the neglected/left and spared/right target regions. As illustrated in **Figure 3B**, a one-way ANOVA on the average benefit of stability in control patients did not reveal a significant difference between correct response times for targets presented in the left vs. right hemifields (*p* = 0.533). To compare the benefit of stability in the neglected/left and spared/right regions between patients and control participants, we next conducted a 2 (side: neglected/Left vs. Spared/Right) within-subjects × 2 (Group: Patient vs. Control) between-subjects ANOVA. There was a main effect of the within-subjects factor of Side [*F*(1,13) = 11.18, *MSE* = 9659.109, *p* = 0.005, $\eta_{p}^{2}$ = 0.462), as well as an interaction between Side and Group [*F*(1,13) = 14.314, MSE = 9659.109, *p* = 0.002, $\eta_{p}^{2}$ = 0.524), but no between-subjects effect of Group (*p* = 0.128). Also as illustrated in **Figure 3B**, non-parametric independent-samples Mann–Whitney *U* tests on the two sets of patient and control data with unequal variances and sample sizes indicated no difference in the benefit of stability between the three patients and twelve controls for targets in the neglected and left regions (*U* = 28, *Z* = -1.443, *p* = 0.18), but did show a significant difference between patients and controls for targets presented in spared and right regions (*U* = 0, *Z* = 2.598, *p* = 0.004).

## DISCUSSION

We investigated how global statistical information affected the abilities of patients with noted attentional deficits to search for a target presented in regions of the visual field where focused attention was found to be absent or severely impaired compared to targets presented in regions where attentional resources appeared intact/similar to control participants. Although the local sizes of all elements changed on each trial, patients tended to correctly discriminate the leftward or rightward tilt of the target Gabor faster in the neglected regions when the stability of the mean size of the background Gabors built over several successive trials as compared to when the mean size of the background Gabors changed and this stable statistical context was broken. On the contrary, the lack of a significant difference between the benefit of statistical stability in control subjects when searching for targets in the left vs. right hemifields provides no evidence to support alternative explanations that hemifield differences in older adults might otherwise account for the observed differences in the effect of statistical stability observed in patients’ neglected compared to spared regions.

A growing body of evidence from studies of normal participants (e.g., Ariely, 2001; Chong and Treisman, 2003,^2005^; Joo et al., 2009; Choo and Franconeri, 2010; Corbett and Oriet, 2011; c.f., Myczek and Simons, 2008) supports proposals that global summary representations do not include precise information about individual elements and are extracted with little or no demands on focal attentional resources. The present results suggest that the stability of such representations facilitates patients in searching for targets in the neglected regions, where attentional resources are compromised. Our findings also extend previous results suggesting patients with attentional deficits are sensitive to the background statistics of visual displays (Demeyere et al., 2008; Leib et al., 2012) by demonstrating that the stability of these statistics can facilitate visual search in regions where patients have difficulty perceiving individual objects. Our results are in-line with a previous report by Saevarsson et al. (2008) that the availability of a stable spatial context improved patients’ performance in neglected regions in a visual search task. Although there was no predictable spatial relationship between the background context and the target in the present study, the repeated statistical context of the background similarly facilitated patients in discriminating the orientation of targets in the neglected regions of the display. Overall, the present results provide converging evidence that patients may rely on statistical information as a preferential strategy to cope with focal attentional impairments and provide novel evidence for a functional role of global statistical representations in visual perception.

Our findings can be interpreted in the context of several visual search models. In terms of the Contextual Guidance model of attention (Torralba et al., 2006) outlined in the Section “Introduction,” patients (and controls) were better able to make global sense of the scene when there was less new information over successive presentations of the same mean size. In other words, when the statistical context became stable over successive displays, it was no longer necessary to parse the display into objects and backgrounds, decreasing demands on attentional resources needed for detailed local processing of task-irrelevant features of the individual contextual elements and increasing resources available for processing the task-relevant feature of the target’s unique orientation. In the framework of Wolfe’s (1994) Guided Search 2.0 model of attention, the statistics of display were automatically extracted and represented in a bottom-up feature map. This map stabilized as the mean size repeated over successive trials, and the unique orientation of the target was more easily discriminated. In general, repeated context reduces the amount of information that must be represented in detail over time. This, in turn, reduces the demands on limited capacity attentional resources, particularly compromised in patients’ neglected regions, and speeds the search process for the uniquely oriented target Gabor.

Interestingly, the cost of stability in patients’ spared regions was not observed for the group of 12 control subjects when the target was presented in the right hemifield. This differential effect of stability in neglected and spared regions may be explained by patients becoming aware of their specific deficits and developing different strategies, both as natural coping mechanisms and through typical therapeutic methods and repeated testing via typical tasks (e.g., cancelation/search) used to assess neglect. For example, patients may have learned to spread attention more diffusely over the entire display, making global statistical properties more available (e.g., Leib et al., 2012). They may also have learned to rely more heavily on focused attention in the spared regions where they were able to perceive individual objects well, and distributed attention over the neglected regions where they were not. In either case, patients may have actually had to devote more attention to processing potential target elements in the spared regions to override this more prominent global representation. These explanations are in-line with Chong and Treisman’s (2005) proposal that mechanisms responsible for representing ensemble statistics operate under conditions of diffuse vs. focused attention. Future work exploring these differences between performance in patients’ neglected and spared regions and their relation to specific therapies and noted search techniques would help to determine whether patients have adopted a modified strategy for searching in the spared compared to neglected regions.

As an alternative explanation to the statistical redundancy of the background mean size allowing for the target to be more quickly localized and discriminated, it is possible that the overall global orientation of the display could be more quickly discerned as rightward or leftward. For example, if patients have learned to distribute attention more broadly across the entire visual field, they are more likely to extract the overall orientation of the elements under such conditions of broadly distributed attention (e.g., Chong and Treisman, 2005). In other words, it is possible that the stability of one ensemble statistic (mean size) allowed for another ensemble statistic (mean orientation) to be more efficiently encoded.

Along these lines, it is also possible that the uniquely oriented target Gabor “popped-out” more when the statistics of the background were repeated, although the reactions times in the present investigation (**Table 2**) were much longer than those typically reported for pop-out search (e.g., Treisman and Gelade, 1980; Duncan and Humphreys, 1989). The idea that distractor features are not represented in detail but instead perceptually summarized is directly in-line with proposals that statistical representations do not include precise information about individual elements and are created in a qualitatively different manner than individual item representations. Perceptual summaries of background/distractor features likely play a role in pop-out effects in search tasks, as well as in other situations, such as in studies of change blindness and under conditions of inattentional blindness. For example, changes that alter the gist or layout of scenes are most often detected in change blindness studies (for reviews, see O’Regan and Noë, 2001; Simons and Rensink, 2005). Furthermore, Moore and Egeth (1997) demonstrated that although observers were not aware of spatial configurations of background elements, these contextual properties nonetheless affected how target objects in the current focus of attention were perceived.

Another important consideration in interpreting the present results is that the global statistics were identical in both neglected and spared regions of patients’ visual fields. Therefore, it could be the case that only the statistical stability of the spared regions contributed to the present results. However, regardless of whether statistics were only represented in the spared regions, stability facilitated search in the neglected regions where focal attentional resources were compromised in a different manner than search in the spared regions. Findings from previous investigations of statistical processing in neglect patients (outlined in the Introduction) showing that statistical properties in the neglected visual field affect statistical processing in the spared regions (e.g., Pavlovskaya et al., 2010; Leib et al., 2012), and proposals that summary representations are calculated based on weighted-averages of items with the sets (e.g., De Fockert and Marchant, 2008; Alvarez, 2011) also support the idea that the statistics of the entire visual display, and not only statistics in patients’ spared regions, were driving the observed effects on search performance.

It has recently been suggested that summary statistical representations have evolved in a complementary manner to focused attention, allowing for the redundancies in the environment to be economically encoded in an average representation and freeing attentional resources for detailed processing of salient local elements (Alvarez, 2011). As the ensemble statistics of the environment are likely to remain stable across saccades, the visual system could rely on these global statistics in attempt to cope with constantly changing local visual stimulation (Melcher and Colby, 2008; Corbett and Melcher, 2014). These average set properties could be computed globally in a manner similar to texture segregation for accurate and rapid organization of the scene into objects and background areas, without the need to focally attend to each individual element in the scene (e.g., Chong and Treisman, 2003). Similarly, summary statistics such as the mean and standard deviation may be used in quickly detecting odd or outlier objects, eliminating the need to search and process individual elements. Assuming that each individual measurement contains some random error, averaging multiple measurements gives a more precise estimate of a set property than sampling individual elements, as random error eventually averages to zero. Indeed, this “power of averaging” could explain Ariely’s (2001) finding of more precise mean estimation and near chance member identification.

A growing body of evidence indicates that some kind of global processing still occurs in neglect patients’ neglected hemifields, despite their lack of local awareness (for review see: Driver and Vuilleumier, 2001). A spared averaging mechanism might provide patients with an advantageous way of coping with visual processing challenges, allowing them to gain a quick, effortless, and meaningful representation of the surrounding environment. Moreover, a preserved ability to build global representations appears to be a reasonable explanation for evidence that patients with severe attentional deficits nonetheless experience a stable and coherent visual world. Representing statistical regularities of the context, rather than individual elements features would provide reliable information to cope with constantly changing visual input.

## Conflict of Interest Statement

The authors declare that the research was conducted in the absence of any commercial or financial relationships that could be construed as a potential conflict of interest.

## Footnotes

1 We split the two blocks of trials performed by control participants and Patient 1 into 4 shorter blocks for Patient 2 and Patient 3. At the outset of the experiment, these two patients required noticeably more time to perform the practice trials, and were much slower in the main blocks of the experiment (Patient 2 ~342 ms slower than Patient 1 on average, and Patient 3 ~ 791 ms slower than Patient 1 on average). This division resulted in approximately equal length blocks for all three patients, but with Patient 2 and Patient 3 performing twice as many blocks as controls and Patient 1 to achieve the same number of total trials. Although we attempted to equalize the total testing time for patients and control participants, patients completed an additional detection task and also required longer breaks between blocks than controls. Therefore, patients required approximately 30–45 min longer than control participants to complete the entire testing session. However, there was no evidence of fatigue, as there was no trend for patients’ response times to increase from the first to the last experimental block.
